# Steroid Mass Spectrometry for the Diagnosis of PCOS

**DOI:** 10.3390/medsci7070078

**Published:** 2019-07-10

**Authors:** Brian Keevil

**Affiliations:** Biochemistry Dept, Wythenshawe Hospital, Manchester University NHS Foundation Trust, Southmoor Rd, Manchester, M23 9LT, UK; brian.keevil@mft.nhs.uk

**Keywords:** androgens, polycystic ovary syndrome (PCOS), liquid chromatography tandem mass spectrometry (LC-MS/MS), testosterone, 11-ketotestosterone

## Abstract

The most appropriate steroids to measure for the diagnosis of hyperandrogenism in polycystic ovary syndrome (PCOS) are still open to debate but should preferably be measured using a high-quality method such as liquid chromatography tandem mass spectrometry (LC-MS/MS). Measurement of testosterone is recommended in all of the current clinical guidelines but other steroids, such as androstenedione and dehydroepiandrosterone sulfate (DHEAS), have also been shown to be useful in diagnosing PCOS and may give additional information on metabolic risk. The 11-oxygenated steroids, and in particular 11KT derived mainly from the adrenal gland, are also increasing in prominence and have been shown to be the dominant androgens in this condition. Polycystic ovary syndrome is a complex syndrome and it is not surprising that each of the clinical phenotypes are associated with different patterns of steroid hormones; it is likely that steroid profiling with LC-MS/MS may be better at identifying hyperandrogensim in each of these phenotypes. Research into PCOS has been hampered by the small sample size of clinical studies previously undertaken and larger studies, preferably using LC-MS/MS profiling of steroids, are needed

## 1. Introduction

The diagnosis of polycystic ovarian syndrome (PCOS) is usually based on the Rotterdam criteria and relies on at least two out of the following: polycystic ovaries, ovulatory dysfunction, and hyperandrogenism [1]. Hyperandrogenism has been evaluated using clinical signs, such as hirsutism, but steroid analysis is now seen to be important in the diagnosis of PCOS because it can provide detailed information on the biochemical basis of hyperandrogenism. For this reason, the latest Endocrine society guidelines for the investigation of hirsutism in women suggest testing for elevated androgens in all women with an elevated hirsutism score [2]. Polycystic ovary syndrome is an important cause of hyperandrogenism and it was the most common finding in 89% of premenopausal and 29% of postmenopausal women presenting with symptoms of hyperandrogenism. However, it is important to identify non-PCOS pathology, such as adrenocortical carcinoma, in these women, and important predictors of non-PCOS pathology were shown to be the pattern and severity of androgen excess measured by steroid analysis [3]. The main source of androgen excess in PCOS remains unclear, with a large body of evidence suggesting the ovaries as the primary source [4], but it is recognised that the adrenal also plays an important role because elevated adrenal androgens, such as dehydroepiandrosterone sulphate (DHEAS), have also been reported in PCOS patients [5]. There is a 20–30% prevalence of elevated DHEAS in women with PCOS, and in approximately 10% of cases, this will be an isolated elevation. However, DHEAS does not always reflect the true output from the adrenal gland and it is known that cases of congenital adrenal hyperplasia (CAH) with increased androgens can be associated with normal DHEAS [6]. 

### 1.1. Which Steroids Do We Measure?

What should be measured to assess hyperandrogenism is still open to debate. Guidelines recommend total testosterone (T), preferably measured using a high-quality method such as liquid chromatography tandem mass spectrometry (LC-MS/MS). Calculated free testosterone is also recommended but its usefulness is disputed by some because circulating values of free testosterone are highly dependent on sex hormone binding globulin (SHBG) levels, which are low in the presence of high insulin levels [7]. However, testosterone is not the only important steroid. Androstenedione (A4) is also now recognised as an important androgen to measure in PCOS [8,9,10,11,12] and it has been shown to give complementary information to testosterone, especially with regard to metabolic risk. It is claimed that A4 is a better marker for PCOS than T but simultaneous measurement of both can be more sensitive for detecting PCOS-related androgen excess and for predicting metabolic risk [13]. Androstenedione has also been shown to be a sensitive marker of hyperandrogenism in women with type 1 diabetes and PCOS, where a similar biochemical phenotype tended to be found in non-diabetic women with PCOS but differed from obese women with PCOS [14].

Recent work has focused on the emergence of other adrenally produced steroids as a major source of androgens in women. The C19 steroid 11-hydroxyandrostenedione (11OHA4), produced by the zona reticularis, is the second-most abundant steroid produced by the adrenal gland but was originally discounted as a steroid of interest because it was thought to have no biological activity [15]. Nevertheless, this view was modified when it was recognised that 11OHA4 is the precursor for the active steroids 11-ketotestosterone (11KT) and 11-ketodihydrotestosterone (11KDHT) [16]. Interest in these steroids has increased dramatically in recent years because of their involvement in not only PCOS but also congenital adrenal hyperplasia and prostate cancer. The 11-oxygenated steroids were first described many years ago but early work showing an elevation of 11OHA4 in patients with PCOS was inconclusive, possibly due to the poor specificity of immunoassays available at the time [17,18].

Poor performance of some of the early immunoassays may undoubtedly have hindered the introduction of these steroids, but recent work using more specific LC-MS/MS methods has shown their true potential clinical value. The importance of 11-oxygenated androgens has since been confirmed by the work of O’Reilly et al. who showed that they represent the majority of circulating androgens in women with PCOS, and like androstenedione, are closely related to metabolic risk markers in these individuals [19].

### 1.2. How Do We Measure Steroids?

High-quality methods are needed to accurately measure steroids and LC-MS/MS has become the method of choice for steroid hormone analysis, mainly because of its ability to multiplex assays, ensure quick analysis times and improve specificity compared to immunoassays [20,21,22]. A further problem with immunoassays is that tests are generally developed for high throughput steroid assays to meet a global market. With this in mind, immunoassay development programmes need to recoup costs in a reasonable time frame and do not usually cater for the smaller volume niche assays, such as A4 and 17-hydroxprogesterone (17OHP), or indeed the11-oxygenated androgens.

Improvements in mass spectrometer design and sample preparation technology have enabled the measurement of many steroids across a wide concentration range. Indeed, standards in steroid analysis have undoubtedly been driven up by the improved specificity of the technique. The improved performance of LC-MS/MS against immunoassays has been recognised by all of the major endocrine journals, and has prompted recommendations for necessary performance criteria that should be met for any method that is used in clinical and non-clinical studies [23,24]. The latest hirsutism guidelines recommend the use of a reliable specialty assay for measuring T [2].

Still, even when a very specific immunoassay is used, some have found that an isolated androgen measurement is unlikely to identify the hyperandrogenic mix that characterises patients with functional ovarian hyperandrogenism and PCOS [25]. Others would go further and argue that LC-MS/MS should preferably be used to identify the androgens instead [26,27].

### 1.3. Liquid Chromatography Tandem Mass Spectrometry

Liquid chromatography (LC) achieves the separation of a mixture of steroids with the use of a column packed with a chemically bonded stationary phase, after which the steroids are identified in the tandem mass spectrometry (MS/MS) detector. Specificity of steroid identification is achieved in the MS/MS detector by measuring fragment ions selectively formed from the fragmentation of their molecular ions. The mass detection process takes place in milliseconds and allows many different steroids to be analysed simultaneously, thus enabling panels of multiple steroids to be measured within one analytical run. Liquid chromatography tandem mass spectrometry is becoming a popular technique in clinical laboratories, and assays for T and A4 are now commonly available in many large centres [26,28,29].

Sample clean up before LC-MS/MS analysis is inevitably required to achieve the removal of interfering substances, reduction of matrix effects and improvement in assay sensitivity. Measurement of steroids is not always straightforward, and in particular, 11KT and 11OHA4 need to be well-separated on the LC column because they share the same molecular weight. Other techniques, such as supercritical fluid chromatography, have also been employed to achieve this critical separation [30]. Whilst incredible chromatographic separation is achievable, almost similar to gas chromatography, the technique is not yet robust enough for use in routine laboratories. Chemical derivatisation of the steroids to improve sensitivity and alter chromatographic behaviour has been favoured by others [31]. This group also showed that the 11-oxygenated steroids do not fall in concentration with age as do other adrenal steroids such as A4 and DHEAS.

We have recently developed a method for T, A4, 11KT, 11OHA4 and 17OHP using acetonitrile in the mobile phase, which confers good resolution between the isobaric steroids, whilst still allowing a fast analysis time of less than five minutes per sample. Non-classical CAH is detected using 17OHP, but care must be taken to ensure that samples are taken in the follicular phase, providing that the patient has normal cycles, because 17OHP, A4 and T can increase significantly mid-cycle [29]. Dehydroepiandrosterone sulphate can also be included in the profile but its very high concentration range in relation to the other steroids often makes it more practical to run as a separate assay.

Multiplexing steroid assays is one of the key strengths of LC-MS/MS, but these assays can be difficult to maintain in a routine clinical laboratory because the complexity and difficulty of preparing calibrators and quality control samples increases as the number of analytes increases. As a consequence, many routine laboratories are now using commercially available material specifically developed for the measurement of steroid panels in LC-MS/MS assays. Service delivery of LC-MS/MS in a routine laboratory has been simplified by the introduction of these easy to use kits (www.Chromsystems.de, www.Recipe.de, Chromsystems, Munich, Germany), and whilst they cater for a wide range of steroids, they do not as yet cater for the 11-oxygenated steroids because of their novelty. Liquid chromatography tandem mass spectrometry has a major role to play in improving the quality of steroid analysis in laboratories, because unlike an immunoassay, it is not subject to differences in antibody specificity, and well-validated methods are generally reproducible between laboratories. Common reference intervals can be developed from shared data as a result of this improved performance, resulting in the major benefit of harmonising result interpretation in different centres. 

### 1.4. Liquid Chromatography Tandem Mass Spectrometry and Polycystic Ovary Syndrome

Lower concentration results are generally found using LC-MS/MS when compared to immunoassay [32], but when comparing LC-MS/MS against immunoassays, it was found that an immunoassay could not discern differences in hyperandrogenism that could be seen with LC-MS/MS. Underestimation of testosterone in immunoassays in these cases was thought to be due to the poor accuracy of these assays in the female range [33].

Liquid chromatography tandem mass spectrometry profiling may offer the best way forward for diagnosing hyperandrogenism but some have found that it provided only limited improvement relative to a T immunoassay [34], and others showed comparable results for LC-MS and immunoassays [10]. However, differences between these studies may have arisen because of different patient populations involving small numbers of patients. 

Diagnosis of PCOS is easier in younger women because androgen levels decrease with age towards menopause, with differences between those with and without PCOS narrowing. Women with PCOS still have elevated androgens post menopause and the tests that best predicted PCOS at all ages were cFT and A4 [35], and this was also true for young girls aged 13–16 years. Steroids measured at baseline and then after one year showed that the major steroids in obese adolescents with PCOS were A4 and T. However, weight loss in the obese PCOS girls was associated with a decrease in T and A4, whereas in obese non-PCOS girls, no significant change was seen [36]. Using a steroid profile to further classify hyperandrogenaemia, this heterogeneity has been described in other women with PCOS, and those women originally identified based on Rotterdam criteria could be re-categorized into eight hyperandrogenic subgroups if T, A4 and FAI were also included [37].

The differences between obese and non-obese women can be marked and a large study exploring differences in the steroid pathways showed that lean women with PCOS had an increased activity of P450c17, P450aro and 3βHSD2, and decreased activity of P450c21. Differences in steroidogenic enzyme activity were found between lean and obese women with PCOS, and adrenal androgen excess was thought to have different roles to play in these women [38]. Cell culture work has since demonstrated that androgen synthesis can be stimulated by increased 17, 20 lyase activity following oxidative stress, which may underlie hyperandrogenism in PCOS [39]. Increased 17,20 lyase activity has also been demonstrated in PCOS patients using gas chromatography mass spectrometry GC-MS urinary metabolomics. While looking for possible biomarkers, it was found that the best combination of steroids was androstanediol, oestriol, 20βOH cortisone and cortisol. Androstanediol, in particular, was a novel finding and it was claimed to show evidence for the role of androgen production by the backdoor steroid pathway in PCOS [40].

Dihydrotestosterone (DHT) is an important androgen that may also be useful in detecting adverse metabolic phenotypes in PCOS [41], but this view has been disputed [34]. 

Androgens have also been measured in saliva using LC-MS/MS and testosterone, A4 and the T/A4 ratio were found to be significantly higher in women with PCOS compared to healthy women. In addition, the T/A4 ratio was associated with glucose intolerance, insulin resistance, metabolic syndrome, obesity and oligo/anovulation [42]. 

Both, 11KT and 11OHA4, as already discussed, have the potential to be major players in the diagnosis of PCOS but there has been little published work on these to date [19], although this may change now that a major contract laboratory in the United States has launched a method for 11KT.

The profiles of these steroids vary among patients with isolated elevations of either classic or 11-oxygenated steroids being seen. In particular, obesity seems to favour accumulation of 11-oxygenated steroids in PCOS, though this needs further verification [43].

## 2. Discussion

Polycystic ovary syndrome is characterised by a combination of hormonal and metabolic disturbances, including hyperandrogenism, but also insulin resistance and glucose intolerance. This is a complex syndrome and it is not surprising that each of the clinical phenotypes are associated with different patterns of steroid hormones. Steroid profiling with LC-MS/MS may be better at identifying hyperandrogenism in each of these phenotypes, but unfortunately many of the reported studies are relatively small. A recent meta-analysis of hyperandrogenism and metabolic parameters in PCOS concluded that there were an insufficient number of studies using LC-MS/MS for measuring androgens to draw any serious conclusions, and that more studies are necessary to assess the androgens’ levels using this accurate method in patients with PCOS [44].

Well-defined reference ranges derived from large numbers of healthy women are desperately needed, and whilst these have been derived for some steroids, they are not currently available for 11-oxygenated steroids [45,46].

Current clinical practice guidelines use relatively old evidence for measuring androgens with data often derived from immunoassays. Androgen profiling using LC-MS/MS is not universally adopted and there is still no consensus as to which androgens are the best to measure and which to include in an ideal test profile. Many laboratories still rely on just testosterone alone to help diagnose PCOS, and because LC-MS/MS is perceived to be relatively expensive compared to immunoassays, many continue to use immunoassays for reasons of cost and convenience. Whilst this may be true for individual steroid assays, as mentioned previously, the great strength of LC-MS/MS is the ability to multiplex many steroids in the same assay at minimal extra cost. Liquid chromatography tandem mass spectrometry actually becomes very cost-effective compared to immunoassays when measuring panels of steroids, and importantly opens up the possibility of measuring steroids currently not available on main immunoassay platforms. Data on the best androgens to use is often conflicting but there is good evidence that measuring testosterone alone will miss a significant number of patients with hyperandrogenism. Consequently, the only way to improve the diagnosis of PCOS will involve measuring androgen profiles and this can only be delivered in a cost-effective way via the use of LC-MS/MS. Those laboratories using LC-MS/MS will probably already measure T and A4 simultaneously within the profile and it would make sense to also include 17OHP and DHEAS to cover the non-PCOS pathology, as already discussed at little extra cost. The 11-oxygenated steroids are exciting new tools for the diagnosis of hyperandrogenism in PCOS and show great promise, but compared to the other androgens, there are as yet few studies assessing their use. It would be straightforward to also include 11KT and 11OHA4 within an LC-MS/MS profile and this combination of steroids may offer the best opportunity to sub-classify this multifaceted syndrome and perhaps point to more targeted treatment strategies, but it should be stressed that research into PCOS has been hampered by the small sample size of clinical studies previously undertaken. 

## 3. Conclusions

Steroid profiling with LC-MS/MS may be better at identifying different phenotypes in PCOS, but larger studies are needed before any valid conclusions on clinical impact can be drawn.
